# Proposing a Model for the National Hemovigilance Information System in Iran

**DOI:** 10.25122/jml-2019-0112

**Published:** 2020

**Authors:** Farkhondeh Asadi, Nahid Ramezanghorbani

**Affiliations:** 1. Department of Health Information Technology and Management, School of Allied Medical Sciences, Shahid Beheshti University of Medical Sciences, Tehran, Iran; 2. Department of Development & Coordination Scientific Information and Publications, Deputy of Research & Technology, Ministry of Health & Medical Education, Tehran, Iran

**Keywords:** Hemovigilance, information system, blood preservation, blood safety, transfusion medicine

## Abstract

The present study aimed to propose a model for the national hemovigilance information system with a database approach, considering the importance and necessity of developing an information system for such a network.

This is an applied, descriptive, and cross-sectional study, which was conducted in 2018. The research population comprised hemovigilance information systems in advanced countries, including the USA, UK, Australia, and France. Data were collected from library sources and the Internet from 2000 to 2018. The proposed model for the national hemovigilance information system was introduced using comparative tables and based on the similarities and differences of systems in the studied countries. The proposed model was then validated using the two-step Delphi technique through a researcher-made questionnaire whose validity was confirmed, and reliability was approved by a Cronbach’s alpha of 94%.

The final model of the national hemovigilance information system comprised five main components: goals, organizations involved in the blood transfusion process, databases of blood transfusion organizations, data transfer flow between the databases of blood transfusion organizations, and transferable datasets, and hemovigilance-related committees. This model was approved by experts with an >85% agreement coefficient.

The national hemovigilance information system with a database approach can improve blood transfusion health by providing access to reliable sources on blood transfusion complications to everyone, especially the medical community. Thus, it is essential to implement this standard accurately and precisely control the practical methods of this process based on international guidelines.

## Introduction

Blood transfusion is the primary concern for medicine. The advancement in the knowledge and awareness on this topic paves the way for the prevention of diseases and the improvement of the treatment process [1]. In the case of situations hazardous for the lives of patients or for stabilizing dangerous health conditions, blood transfusion is essential [2].

The process of blood transfusion comprises various clinical and laboratory services; therefore, all the steps in this process must be followed with precision, efficiency, and reliability in order to prevent unwanted complications [1]. Blood transfusion risks may lead to mortality, life-threatening situations, or disability in donors or recipients [3]. These reactions not only lead to severe discomfort in patients but also impose a heavy burden on the healthcare system [1,4]. Today, with the advancement in technology, the process of blood transfusion has improved. Still, the incidence of complications resulting from human error, ABO incompatibility, bacterial infection, and immunological phenomena remains the main concerns [5]. Accordingly, monitoring methods and patient safety requirements must be considered throughout the blood transfusion process as a hemovigilance system. In fact, this system analyzes blood transfusion-related reactions and decreases blood product-related risks so that complications are reported faster and more accurate [6].

A hemovigilance system is a tool for quality improvement in the blood transfusion process, mainly focusing on safety [3,7]. This system must be completely merged and integrated into the quality systems of all institutions in charge of blood and blood product supply chains, in order to ensure the safety of donors and recipients. This system looks like a cycle of continuous quality improvement, showing a set of activities in each step of the transfusion process as well as every organization in charge of part of the chain [8]. In this system, information plays a vital role in accurately reporting transfusion-relation complications, timely documentation, and error prevention in various steps of the process [9].

The World Health Organization published in 2007 the standards on the blood transfusion process. Based on this report, the global database on blood safety, 42 countries have implemented national hemovigilance systems under different titles, e.g., the Haemovigilance Advisory Committee (HAC) in Australia in 2008 [10], the French National Agency for Medicines and Health Products Safety (ANSM) in France in 1993 [11], Serious Hazards of Transfusion (SHOT) in the UK in 1996 [12], and the AABB Center for Patient Safety in the US in 2006 [9]. The goal of all these systems is collecting and reporting the complications and unwanted reactions in the process of blood transfusion [13, 14]. These reports prove that “using blood”, and “hemovigilance” are health-related concerns around the world [5].

As Iran is a member of the International Haemovigilance Network, due to the importance of designing such an information system for this network, and since no comprehensive study has been conducted [15] on a national hemovigilance information system in Iran, the present study aimed to propose a model for national hemovigilance information system with a database approach.

## Material and Methods

The present applied, descriptive, and cross-sectional study was conducted in the following three steps:

1. Review of the literature
  In this step, the required data were collected based on a review of the literature on a hemovigilance information system and its main components in different countries, including the US, UK, Australia, and France, in Ovid, Scopus, PubMed, ScienceDirect, and Google Scholar databases from 2000 to 2018 using the following keywords: National Hemovigillance System, Hemovigilance System, Hemovigilance Network, Hemovigilance Information System, and Transfusion blood system.
2. Developing a model for hemovigilance information system in Iran
  In this step, organizations in charge of blood transfusion and hemovigilance system in Iran were identified, and their work process was examined. Afterward, based on the results of the literature review and the organization and structure of the Iranian Blood Transfusion Organization (IBTO), a model for the national hemovigilance information system was developed.
3. Model validation
  Validation was performed using the Delphi technique in two steps. First, a researcher-made questionnaire, including the demographic information of system users and the model of axes, was developed. The questionnaire was given to six experts (three health information management experts and three hematology experts), and their opinions were sought to check the validity. Moreover, Cronbach’s alpha was calculated to determine the reliability of the questionnaire, which equaled 94%. As the value was above 0.7, the questionnaire was found to have high reliability. Then, the final model was given to 13 hematology experts at medical universities in Tehran, as well as the IBTO. Expert opinions on the main components of this model were collected, and the agreement coefficient of 75% was set as the criterion for model acceptance. Next, expert opinions were applied to the proposed model. In the second step of the Delphi technique, an expert panel with six hematology and health information management experts was formed in order to finalize the proposed model. Descriptive statistics were used to analyze the data on the final model confirmation.

## Results

The review of the literature revealed that the goal shared by all countries with hemovigilance networks or systems included monitoring transfusion-related complications and reactions and enhancing quality and patient safety by improving the reporting system. Accordingly, the most important components of the hemovigilance information system were determined to be the definition and specifications of organizations involving in the hemovigilance system, databases, and data transfer flow in each database of the noted organizations.

The proposed model for the national hemovigilance information system with a database approach in Iran comprised five main components:

1. Goals of the national hemovigilance information system
2. Organizations involved in the blood transfusion process
3. Databases of blood transfusion organizations
4. Data transfer flow and transferable datasets
5. Hemovigilance-related committees

Major organizations involved in the blood transfusion process included blood donation and transfusion centers, blood transfusion organizations in provinces, the IBTO, and the Ministry of Health and Medical Education (MoHME).

Data transfer in the process of blood transfusion has been planned at the city, province, and national levels. There are centers for donating and receiving blood at the city level, including blood transfusion organizations at the province level, blood donation centers, and mobile centers for blood donation. The data transfer process was designed as follows: after blood donation, in case of any complications in the donor, this event is immediately reported to the hemovigilance committees at the province level in the blood transfusion organization. Centers receiving blood donation include hospitals and clinics for special diseases (thalassemia) in which blood is transfused. In hospitals, complications are immediately reported to the hemovigilance chief physician. Using the national hemovigilance information system, a set of data elements (Table 1) is recorded in this system, reports are prepared, and measures are taken by allowing stakeholders access information.

**Table 1:** Set of data elements in the national hemovigilance information system.

Data elements
Patients’ demographic characteristics
Healthcare events and side-events
Intensity of outcome
Blood transfusion measures
Examination of results
Cooperating factors or agents
Type of product
Measures
Transfused volume and the time interval since the onset of transfusion and the event
Patients’ clinical condition at the onset of transfusion and during an event
Ability to trace each part of the blood transfusion process
Examination of transfusion-related complications by the hemovigilance chief physician

Moreover, a hemovigilance committee is formed in hospitals. If the complication turns out to be related to the transfusion process, this problem is reported to the hemovigilance committee at the province level.

Based on organizations and activities related to the blood transfusion process, hemovigilance committees are formed at different levels consist of hospital, provenance, and national level committees.

### Validation of the Proposed Model for the National Hemovigilance Information System

#### Findings of the first step of the Delphi technique

Findings of model validation indicated that all major axes were completely accepted by experts. However, there were some disagreements on several sub-criteria of the major axes. In cases where the agreement coefficient was <75%, those criteria were removed. Furthermore, some sub-criteria were suggested for addition. These suggestions were discussed by experts in the second step of the Delphi technique. The results are presented below.

The suggested goals for the national hemovigilance information system, including policy-making and better allocation of financial resources, health planning for blood donors and recipients, evidence-based care planning, monitoring of blood and blood product supply chain, preparing statistical reports on transfusion-related unwanted events, blood processing and transfusion, and measures for preventing complications were agreed upon by all experts (100%).

The formation of hemovigilance committees at the city, province, and national level was agreed by all experts. Also, all experts agreed with sending the data online (Table 2).

**Table 2:** Frequency percentage distribution of expert opinions on other components of the system.

**Frequency Percentage Distribution of Expert Opinions on Organizations Involved in the National Hemovigilance Information System**							
**Expert opinion** **Cooperating organizations**		**Agree**		**No opinion**		**Disagree**	
		**Frequency distribution**	**Percentage**	**Frequency distribution**	**Percentage**	**Frequency distribution**	**Percentage**
**1**	IBTO	13	100%				
**2**	Treatment Vice-Chancellorship of MoHME	12	92.3%			1	7.6%
**3**	Health Vice-Chancellorship of MoHME	13	100%				
**4**	Food and Drug Vice-Chancellorship of MoHME	4	30.7%	7	53.8%	2	15.3%
**5**	Universities of medical sciences	12	92.3%	1	10%		
**6**	Hospitals	13	100%				
**7**	Blood and blood product research centers	13	100%				
**8**	Blood specialized associations	11	84.6%	2	15.3%		
**9**	Iranian Society of Pathology	6	46.1%	5	38.4%	2	15.3%
**10**	Iranian Pediatric Hematology & Oncology	13	100%				
**11**	Iranian Scientific Association of Pediatric Hematology & Oncology,	13	100%				
**12**	Iranian Pediatric Hematology & Oncology Society	9	69.2%	3	23.7%	1	7.6%
**13**	Iranian Association of Clinical Laboratory Doctors	10	76.9%	3	23.07%		
**14**	Iranian Scientific Association of Clinical Laboratory	7	53.8%	6	46.1%		
**15**	Iranian Blood Transfusion Association	13	100%				
**16**	Research center of IBTO	12	92.3%	1	7.6%		
**17**	Iranian Laboratory Hematology Association	13	100%				

Findings in this table show that the IBTO, Health Vice-Chancellorship of the MoHME, hospitals, blood and blood product research centers, Iranian Society of Medical Oncology and Hematology, Iranian Scientific Association of Pediatric Hematology & Oncology, Iranian Blood Transfusion Association, and Iranian Laboratory Hematology Association were approved by experts (100%). Nevertheless, the Food and Drug Vice-Chancellorship of the MoHME, Iranian Society of Pathology, Iranian Pediatric Hematology & Oncology Society, and Iranian Scientific Association of Clinical Laboratory were eliminated as they scored below 75% (Table 3).

**Table 3:** Frequency Percentage Distribution of Expert Opinion on the Members of the Hemovigilance Hospital Committee.

**Expert opinion** **System’s hospital committee members**		**Agree**		**No opinion**		**Disagree**	
		**Frequency distribution**	**Percentage**	**Frequency distribution**	**Percentage**	**Frequency distribution**	**Percentage**
**1**	Head of the medical center of his/her deputy as the head	12	92.3%	1	7.6%		
**2**	Head of the medical center	12	92.3%	1	7.6%		
**3**	Technical official of the medical center	13	100%				
**4**	Heads of main blood consuming wards, e.g. ICU, internal medicine, surgery, anesthesia, pediatrics, gynecology, or others wards as selected by the technical official of the medical center	13	100%				
**5**	Technical official of the laboratory	13	100%				
**6**	Blood surveillance head physician	13	100%				
**7**	Blood transfusion and management consulting physician	12	92.3%			1	7.6%
**8**	Blood bank official at the medical center	13	100%				
**9**	Chief nurse (head of nursing services) at the medical center	13	100%				
**10**	Information management official at the medical center	8	61.5%	4	30.7%	1	7.6%
**11**	Head of the operating room at the medical center	7	53.8%	6	46.1%		
**12**	A representative of the financial affairs unit	4	30.7%	8	61.5%	1	7.6%

It is evident that that, except for the head of the information management unit, the operating room supervisor, and a representative from the financial affairs unit, the other members were approved by experts (<92%). Therefore, the remaining sub-criteria were eliminated from the final model. It was also suggested that the following be added to the hospital committee: educational supervisor, head of quality improvement unit, patient safety contact, hemovigilance nurse, treatment deputy, and legal medicine expert.

Also, findings show that all members were approved by scores of >84%. It was also suggested that the following should be added to province-level committees: patient safety intermediary, hemovigilance chief nurse, a representative of the province-level laboratory affairs unit, and medical associations (especially emergency medicine, surgery, gynecology, and oncology) (Table 4).

**Table 4:** Frequency Percentage Distribution of Expert Opinion on the Members of the National Hemovigilance Hospital Committee.

**Expert opinion** **System’s national committee members**		**Agree**		**No opinion**		**Disagree**	
		**Frequency distribution**	**Percentage**	**Frequency distribution**	**Percentage**	**Frequency distribution**	**Percentage**
**1**	Minister of MoHME as the head of the work-group	12	92.3%	1	7.6%		
**2**	Vice-chancellor of the IBTO as the secretary	13	100%				
**3**	Treatment vice-chancellor of MoHME	12	92.3%	1	7.6%		
**4**	Chancellors of universities of medical sciences	11	84.6%	2	15.3%		
**5**	General manager of the Health Reference Laboratory of the MoHME	12	92.3%	1	7.6%		
**6**	General manager of the Medical Supervision and Accreditation of MoHME	11	84.6%	2	15.3%		
**7**	Head of the Iran Medical Council	11	84.6%	2	15.3%		
**8**	CEO of Social Security Organization	8	61.5%	5	38.4%		
**9**	Chief of clinic of General Staff of the Armed Forces of the Islamic Republic of Iran	9	69.2%	4	30.7%		
**10**	CEO of the Iran Health Insurance Organization	8	61.5%	5	38.4%		
**11**	Technical and novel technology deputy of the IBTO	7	53.8%	6	46.1%		
**12**	Head of the hemovigilance and patient blood management office of the IBTO	11	84.6%	2	15.3%		

Based on Table 4, the majority of members were approved by experts upon scoring <84%. On the other hand, the following were eliminated from the final model due to scoring <75%: CEO of the Iran Health Insurance Organization, Technical and Novel Technology Deputy of the IBTO, and the head of the Hemovigilance and Patient Blood Management Office of the IBTO.

#### Findings of the second step of the Delphi technique

**Figure 1:**
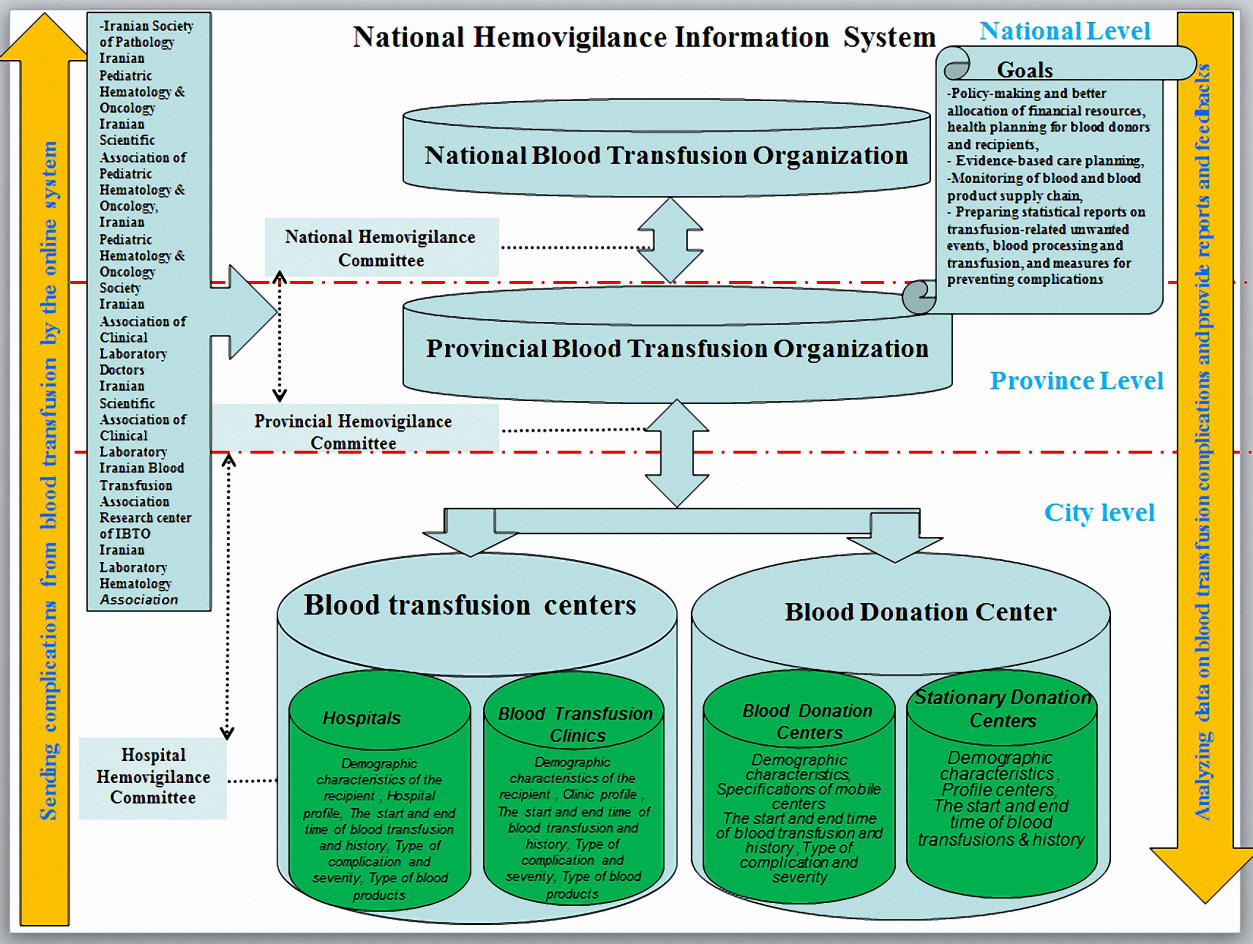
Diagram of the national hemovigilance information system.

In the second step of the Delphi technique, the proposed model was finalized, and all experts completely agreed (100%) with the suggestions added to the model (Figure 1).

The goal shared by all countries with hemovigilance networks or systems is monitoring transfusion-related complications and reactions and enhancing the quality and patient safety by improving the reporting system. Accordingly, the proposed model for the national hemovigilance information system with a database approach in Iran comprised the five components of goals, organizations involved in the blood transfusion process and their databases, data transfer flow, transferable datasets, and hemovigilance-related committees. Organizations participating in this system included the IBTO, Health Vice-Chancellorship of the MoHME, hospitals, blood and blood product research centers, Iranian Society of Medical Oncology and Hematology, Iranian Scientific Association of Pediatric Hematology & Oncology, Iranian Blood Transfusion Association, and Iranian Laboratory Hematology Association. Similarly, the Australian National Blood Authority cooperates with organizations and committees such as the Peripheral blood mononuclear-stem cell (PBMSC) monitoring committee, the National Immunoglobulin Governance Advisory Committee (NIGAC) and the Australian Haemophilia Centre Directors’ Organisation (AHCDO). Associations cooperating with this system include the Australian Association of Pathology Practices (AAPP), Australia and New Zealand Society of Blood Transfusion (ANZSBT), and Australian Private Hospitals Associations (APHA). It is noteworthy that the Food and Drug Administration (FDA) in the US [16] and the Australian Red Cross and the Therapeutic Goods Administration (TG) in Australia also cooperate with the hemovigilance system [17].

In the study conducted by Ganz and Wu [18], the 2016 Australian Haemovigilance Report [19], and the study by Sandid et al. [20], the models of the national hemovigilance system in Canada, Australia, and France with the data transfer process have been developed at a hospital, province, and national level. The present study confirmed this process at the mentioned levels. In Canada, two work-groups of Surveillance and Epidemiology of Transfusion (SET) and Blood-Borne Pathogens Division are in charge of planning and implementing this system. Moreover, the Transfusion Transmitted Injuries Surveillance System (TTISS) is supported by the national work-group. Currently, the Canadian national hemovigilance system consists of five components: TTISS; transfusion errors surveillance; tissue, cell, and organ surveillance system; targeted surveillance for emerging pathogens; and targeted surveillance for the high-risk population [18, 21].

In the present study, the following data elements were included: patients’ demographic characteristics, healthcare events and side-events, the intensity of outcome, blood transfusion measures, the examination of results, type of product, transfused volume and time interval since the onset of transfusion until the event, patient’s clinical condition at the onset of transfusion and during the event, ability to trace each part of the transfusion process, examining whether complications are due to transfusion, and the responsibilities of the hemovigilance chief physician.

Similarly, the study by Hillis et al. [22] confirmed the data elements of the present study while discussing other elements, including signs and symptoms, laboratory results (vital signs, X-ray results, blood burden evidence), blood transfusion characteristics (time and data of events and report, transfusion location, medications, blood components, treatments, hospital measures, transfusion-related blood infection report), judgment on negative reactions to blood transfusion (diagnostic classification of transfusion complications, level and intensity of complications, complications outcome, the relationship between blood transfusion and mortality in the recipient), and research (hospital measures, medical follow-up, treatment or prevention).

Moreover, in the present study, reports are daily and electronically sent, and the method of reporting is based on pre-determined reporting formats (monthly, seasonal, and annual) at different levels (national, regional, province, and city) based on the information needs of stakeholders. Similarly, in the study by Sandid et al. [23] and Liang et al. [24], the e-FIT system in France monitoring blood transfusion collects the reports on blood-related data and recipients and calculates the incidence and prevalence of events in 100,000 blood donors or blood contents annually.

Information is distributed and published using websites, software programs, fax, and automation in the proposed model for the hemovigilance information system in the present study. Consistent with the study by Vanagas et al. [25], the information system of 23 hemovigilance organizations was examined. These organizations consider web-based information systems to be a solution for blood monitoring and preservation. However, due to the lack of comprehensive and compatible data, information distribution is performed in a non-electronic manner.

## Conclusion

The national hemovigilance information system with a database approach can improve blood transfusion health by providing access to reliable sources on blood transfusion complications to everyone, especially the medical community. Thus, it is essential to accurately implement this standard and precisely control the practical methods of this process based on international guidelines.

## Conflict of Interest

The authors declare that there is no conflict of interest.
